# Cervicogenic Somatic Tinnitus: A Narrative Review Exploring Non-otologic Causes

**DOI:** 10.7759/cureus.65476

**Published:** 2024-07-26

**Authors:** Smriti Wadhwa, Shraddha Jain, Nimisha Patil, Shyam Jungade

**Affiliations:** 1 Otolaryngology - Head and Neck Surgery, Jawaharlal Nehru Medical College, Datta Meghe Institute of Higher Education and Research, Wardha, IND; 2 Community Health Physiotherapy, Maharashtra Institute of Physiotherapy, Latur, IND

**Keywords:** multidisciplinary approach, manual therapy, somatosensory modulation, dorsal cochlear nucleus, cervicogenic somatic tinnitus

## Abstract

Cervicogenic somatic tinnitus (CST) is a subgroup of somatosensory tinnitus that arises from altered sensory input from the cervical spine due to changes in anatomical and physiological functions. Unlike primary tinnitus, usually caused by auditory system issues, CST is due to somatosensory disruptions from the cervical region. Conditions such as degenerative disc disease, cervical spondylosis, whiplash injuries, and neck muscle stress or spasms are commonly associated with CST. The pathophysiology of CST involves complex interactions between the cervical spine’s somatosensory inputs and central auditory pathways, particularly affecting the dorsal cochlear nucleus (DCN) in the brainstem, leading to enhanced excitability and synaptic reorganization, giving rise to tinnitus. Accurate diagnosis and management of CST require a comprehensive approach, including patient history, physical examination, audiological assessments, and imaging studies. Treatment strategies encompass physical therapy, medications, interventional procedures, and complementary therapies, aiming to reduce tinnitus perception, alleviate neck dysfunction, and improve overall quality of life. Emerging therapies, such as neuromodulation and regenerative medicine, show promise in further improving CST management. This multidisciplinary approach highlights the importance of addressing both musculoskeletal and auditory health in the effective treatment of CST.

## Introduction and background

Cervicogenic somatic tinnitus (CST) is a specific form of somatosensory tinnitus that arises from altered sensory input originating in the cervical spine due to changes in anatomical and physiological functions. This is unlike primary tinnitus, which is usually associated with hearing loss or other auditory disorders. These disruptions can modify the sensory information processed by the auditory pathways, leading to the perception of tinnitus. CST is frequently associated with conditions such as degenerative diseases, cervical spondylosis, whiplash injuries, and neck muscle spasms.

The pathophysiology of CST involves a complex interaction between the inputs from the cervical spine and the central auditory pathways. Forceful contractions of the jaw and neck muscles can influence the psychoacoustic characteristics of tinnitus [1]. The involvement of the dorsal root ganglia of the second, seventh, and eighth cervical nerves, along with the trigeminal ganglion, highlights the intricate connections with the auditory system. Changes in brainstem structures related to hearing, induced by altered cervical input, are likely contributors to CST. Notably, the dorsal cochlear nucleus (DCN) in the brainstem, which receives input from the auditory nerve, plays a crucial role in the development of tinnitus [2]. Prolonged aberrant somatosensory input from the cervical spine can lead to enhanced excitability and reorganization of synapses in the DCN, sustaining tinnitus perception even without ongoing cervical spine impairment [3,4]. 

The incidence and prevalence of cervicogenic tinnitus are not well documented in large population studies. However, smaller studies and clinical observations suggest it is a recognized but relatively uncommon condition [1]. Therefore, understanding the definition and mechanisms of CST is essential for accurate diagnosis and effective treatment. The interplay between the auditory system and the cervical spine underscores the importance of a multidisciplinary approach that addresses musculoskeletal and auditory health. With ongoing research into these pathways, the ability to diagnose and manage CST will continue to improve, offering hope for better patient outcomes [1].

## Review

Pathophysiology

**Figure 1:**
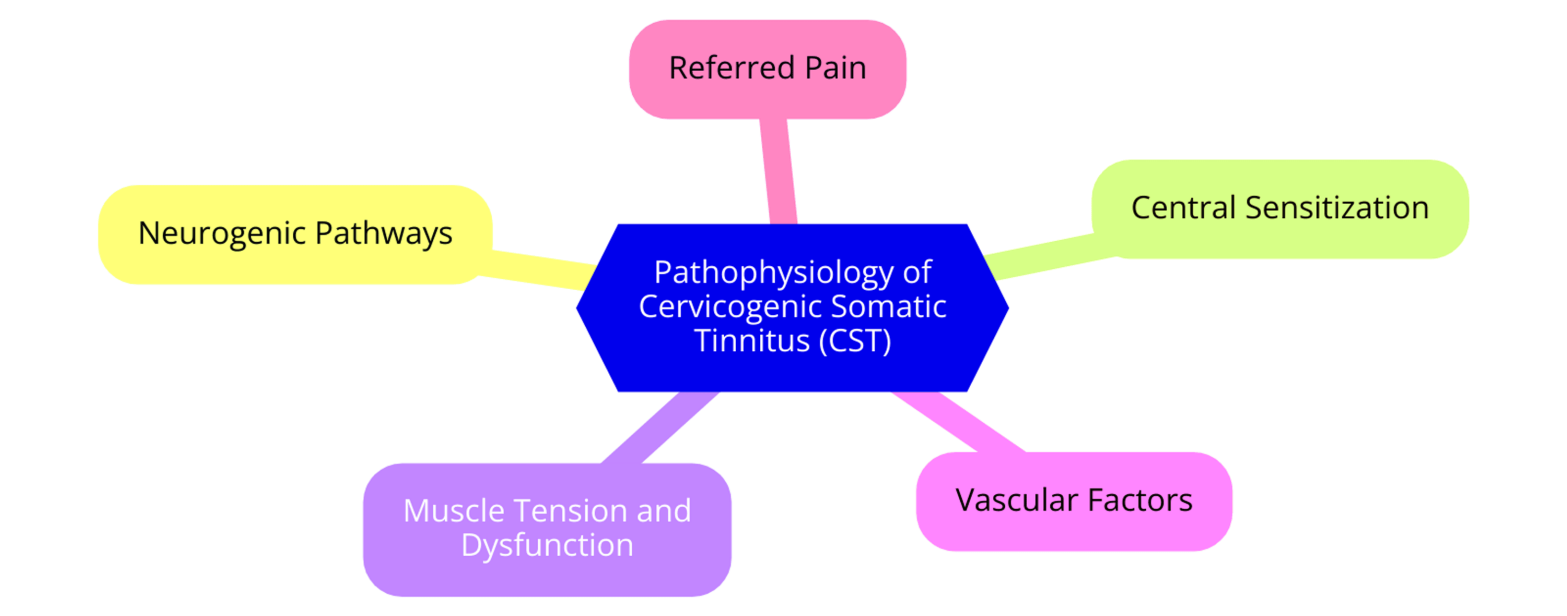
The pathophysiology of cervicogenic somatic tinnitus (CST) Image credit: Dr Smriti Wadhwa

The pathophysiology of CST is complex and involves the interplay between the cervical spine's somatosensory inputs and the central auditory pathways. It is characterized by forceful contractions of the jaw and neck muscles that modulate its psychoacoustic attributes [5]. The involvement of the dorsal root ganglia of the second, seventh, and eighth cervical nerves, along with the trigeminal ganglion, establishes connections with the auditory system. Changes in the brainstem structures related to hearing, induced by alterations in cervical input, likely contribute to CST. It can be considered a form of somatic tinnitus [6]. The DCN in the brainstem receives input from the auditory nerve and plays a crucial role in the development of tinnitus [7]. Hypotheses suggest that proprioceptive and nociceptive inputs from the cervical spine influence neuronal regions in the brainstem related to hearing, with the DCN being inhibited due to cuneate nucleus activity [8]. Associations have been proposed between tinnitus and cervical spine pathologies, neck myofascial tightness, or cervical neck instability [9]. Prolonged aberrant somatosensory input from the cervical spine can lead to enhanced excitability and reorganization of synapses in the DCN, which can sustain tinnitus perception even in the absence of persistent cervical spine impairment, which can then lead to central sensitization. In this condition, the central nervous system becomes more sensitive to external stimuli [10]. This interaction, involving both somatosensory and auditory systems, may contribute to the manifestation of somatosensory tinnitus, an entity not widely recognized among otolaryngologists [8,11]. Additionally, cervical spine abnormalities may affect blood flow to the auditory pathways. For instance, temporary ischemia in the brain areas responsible for processing auditory information can result from spasms or vertebral artery compression. This vascular component may worsen the symptoms of tinnitus [12]. Accurate diagnosis and successful treatment of CST depend on understanding its definition and mechanism. The complex relationships between the auditory system and the cervical spine highlight the significance of addressing CST through a multidisciplinary strategy prioritizing musculoskeletal and auditory health. Our capacity to identify and manage this complicated illness will continue to improve with additional investigation into these pathways. The pathophysiology of CST is shown in Figure 1. 

Diagnosis of CST

CST must be diagnosed using a comprehensive and methodical approach to distinguish it from other types of tinnitus and determine the underlying cervical spine problems causing the condition. The first step is to obtain a thorough patient history, focusing on the tinnitus's onset, course, and features, along with any related neck discomfort, stiffness, trauma, or past cervical spine diseases. It is important to determine from patients what activities, such as certain neck postures or movements, can make their tinnitus worse or better. A thorough examination of the cervical spine is part of the physical examination, aiming to detect joint dysfunction, muscular tension, pain, and limits in range of motion. Assessing the patient's posture and feeling for trigger points or spasms in the neck muscles are important tasks for the clinician. A neurological examination is also necessary to identify any sensory or motor deficiencies associated with cervical spine pathology [13]. Further diagnostic insights can be obtained by performing provocative tests, such as cervical spine rotation, extension, and flexion, to see if these movements replicate or change the tinnitus [14].

Evaluations of auditory function are essential in differentiating CST from other forms of tinnitus. Pure tone audiometry detects any hearing loss that can be linked to tinnitus and assesses hearing sensitivity. To rule out middle ear disorders, tympanometry evaluates middle ear function. Otoacoustic emissions (OAEs) assess cochlear (outer hair cell) function and provide information about the integrity of the peripheral auditory system. Furthermore, testing for the auditory brainstem response (ABR) evaluates the soundness of the auditory pathways leading up to the brainstem, which aids in ruling out retro-cochlear diseases. The diagnosis of CST is mostly dependent on imaging studies. The cervical spine's soft tissues, such as the intervertebral discs, spinal cord, and nerve roots, can be seen with magnetic resonance imaging (MRI), which detect structural anomalies such as disc herniations or degenerative alterations. To identify skeletal abnormalities such as facet joint arthrosis and cervical spine osteophytes, computed tomography (CT) scans offer detailed images of bony structures. Cervical spine X-rays can detect large structural anomalies such as spondylosis, degenerative disc disease, and misalignments [15].

Electrophysiological examinations evaluate nerve conduction and muscle electrical activity. Examples of these procedures are nerve conduction studies (NCS) and electromyography (EMG). These tests help diagnose peripheral neuropathies or nerve root irritation that may cause tinnitus and neck pain. Muscle stiffness can be measured, among others, via myotonometry and elastography [16]. To identify the cause of neck pain and its relation to tinnitus, specialized examinations such as cervical provocation tests use selective nerve root blocks or facet joint injections under fluoroscopy. If cervical facet joints are involved in tinnitus, diagnostic facet joint blocks may be able to aid. In addition to offering more details on vestibular involvement in tinnitus, vestibular evoked myogenic potentials (VEMPs) evaluate the saccule and inferior vestibular nerve [17]. Blood tests can be done to rule out systemic diseases like autoimmune or inflammatory disorders that may be linked to tinnitus and cervical spine problems. Accurately identifying CST requires a thorough diagnostic strategy that includes imaging examinations, specialized diagnostics, audiological assessments, and clinical evaluations.

Treatment of CST

CST must be managed using a multimodal strategy that considers the auditory symptoms caused by the cervical spine disease and the pathology itself. A multidisciplinary team is frequently responsible for coordinating physical therapy, pharmaceuticals, interventional procedures, and complementary therapies in an effective treatment regimen. The main objectives are reducing tinnitus perception, relieving neck dysfunction, and enhancing patients' overall quality of life. The cornerstone of CST management is physical therapy, with the main goals of relieving musculoskeletal tension and restoring the cervical spine to its natural function. By reducing muscular tension and cervical spine stiffness, techniques such as joint mobilization, manipulation, and soft tissue massage might lessen aberrant somatosensory input to the auditory pathways [18]. In physical rehabilitation, customized workout regimens are designed to increase range of motion, strengthen neck muscles, and improve posture. Some specific exercises include stretching, isometric workouts, and cervical spine stabilization exercises. The long-term management of cervical disc degeneration requires maintaining the health of the cervical spine, which can be achieved by teaching patients about ergonomics and good posture [19]. Cervical spine diseases can be treated with medications to reduce discomfort and inflammation. Nonsteroidal anti-inflammatory drugs (NSAIDs), such as ibuprofen and naproxen, can lessen pain and inflammation in the cervical area, which helps to mitigate one of the reasons that lead to cervical sciatic pain. Muscle relaxants, like cyclobenzaprine and tizanidine, can potentially lessen aberrant somatosensory input to the auditory system by easing neck tension and muscle spasms. Acute or persistent neck discomfort can be treated with analgesics like paracetamol or ibuprofen [20].

Interventional procedures may provide significant relief for patients who do not respond to conservative treatments. Cervical epidural steroids deliver anti-inflammatory steroids directly to the cervical spine, reducing inflammation and pain, and alleviating tinnitus symptoms linked to cervical pathology. Selective nerve blocks can relieve pain by interrupting pain signals from the cervical spine to the auditory pathways, thereby reducing abnormal sensory input [21]. Alternative medicine, involving several complementary therapies, could further assist with CST management. Through neuro-modulatory processes, acupuncture may improve the experience of tinnitus and help lessen discomfort and muscular tension in the cervical region. Chiropractic adjustments work by treating spinal misalignments and enhancing cervical spine function. Therapeutic massage can help reduce neck stress and enhance circulation, contributing to overall symptom reduction [22]. Recent advancements have introduced new therapeutic approaches that show promise in controlling CST. Neuromodulation techniques, such as repetitive transcranial magnetic stimulation (rTMS) and transcutaneous electrical nerve stimulation (TENS), attempt to alter brain activity and lessen tinnitus perception [23]. Newer regenerative therapeutic approaches, including stem cell treatment and platelet-rich plasma (PRP) injections, are being explored for their potential to repair and regenerate damaged cervical spine tissues, potentially reducing CST symptoms. Botulinum toxin injections into cervical muscles can reduce muscle spasticity and pain, which may help alleviate CST [24]. Cognitive behavioral therapy (CBT) is useful when tinnitus has psychological effects, as it helps patients cope with the anxiety they experience due to tinnitus [25].

Manual therapy for CST

A key component of treating CST is manual therapy, which aims to relieve musculoskeletal stress and return the cervical spine to normal function. Understanding the underlying somatosensory mechanisms of the proprioceptive and nociceptive innervated structures in the head and neck regions (joints, muscles, ligaments, fasciae, and spinal and peripheral nerves), as well as dysregulated nociceptive processing in the central nervous system regarding the hypersensitivity of the DCN, is the first step toward offering manual therapy as a treatment option [26]. Using hands-on approaches, certified physical therapists or chiropractors address cervical spine dysfunctions and related soft tissue disorders in this therapy approach. Reducing aberrant somatosensory input from the cervical region to the auditory pathways is manual therapy's main objective, which helps mitigate tinnitus symptoms. Joint mobilization and manipulation can enhance joint function, lessen pain, and improve mobility; joint mobilization entails passively moving particular cervical joints within their natural range of motion. Techniques include low-amplitude, high-velocity thrusts, and low-velocity oscillatory movements. Chiropractors frequently use high-velocity thrusts to manipulate the spine, targeting a particular cervical joint. By using this method, joint misalignments can be corrected, nerve irritation can be decreased, and normal joint mechanics can be restored [27].

Soft tissues, like the muscles, fascia, and connective tissues surrounding the cervical spine, are the focus of soft tissue mobilization treatments. These methods, which include myofascial release, trigger point therapy, and deep tissue massage, are intended to soothe pain, promote better circulation, and lessen muscle tension. These approaches reduce aberrant somatosensory inputs that may worsen tinnitus by targeting tight or spasmodic muscles [28]. In muscle energy techniques (METs), the patient voluntarily contracts particular muscles in opposition to a counterforce that the therapist applies. This method helps increase joint range of motion, extend shortened muscles, and improve muscular function. Correcting muscular imbalances and returning the cervical spine's biomechanics to normal can be very successful with METs [29].

In cervical traction, a mild pulling force is applied to the neck to decompress the cervical vertebrae and lessen pressure on the spinal nerves. This method can enhance cervical spine alignment and reduce pain. A therapist can apply traction manually or with mechanical tools and should repeatedly perform very gentle, low-velocity passive articular movements in the spinal joints (including the pelvic joints) and lengthening contraction exercises for the craniocervical muscles [30]. These techniques can dramatically reduce neck pain and discomfort. It helps restore the normal function of the cervical spine by improving the mobility of the joints and soft tissues, which reduces aberrant sensory inputs to the auditory pathways. It is also an excellent means of reducing cervical tension and muscle spasms, which are typical causes of CST. Fewer abnormal signals are produced by relaxed muscles, which may impact the auditory system. Better circulation, brought on by manual therapy, supports the general health of the cervical spine by removing metabolic waste products and nourishing tissues. It can have a neuro-modulatory effect by adjusting sensory inputs at the cervical level, which may lessen the severity and frequency of tinnitus [31].

Even if manual treatment has a lot of potential benefits, it is contraindicated in patients with severe osteoporosis, rheumatoid arthritis, and cervical spine fractures. Therefore, each patient should be carefully evaluated for the safety and suitability of manual therapy [32]. Manual therapy is essential for treating cervical spine dysfunctions that lead to aberrant somatosensory input in cases of CST. Manual therapy can alleviate discomfort, increase cervical mobility, lessen muscle tension, and improve the general health of the cervical spine using methods such as joint mobilization, soft tissue mobilization, METs, and cervical traction. Manual therapy has the potential to greatly enhance the quality of life and lessen tinnitus symptoms for individuals with CST when included in an all-encompassing treatment approach. Emerging treatments in regenerative medicine also hold promise for further improving the management of CST. By combining these therapies, clinicians can provide holistic and effective care, reducing tinnitus symptoms and improving the quality of life for patients with CST.

## Conclusions

CST is a complicated illness resulting from aberrant somatosensory input influencing auditory perception in cervical spine problems. Complex neuroanatomical pathways that link the cervical spine to the auditory system are involved in the pathophysiology, and important roles are played by muscular stress, joint dysfunction, and neural plasticity. Accurate diagnosis and a combination of therapy specifically designed to address cervical spine pathology and tinnitus symptoms are essential components of a complete, multidisciplinary approach to the effective management of CST. Diagnostic testing is crucial to distinguish CST from other forms of tinnitus and find underlying cervical problems. A thorough patient history, physical examination, audiological exams, and imaging procedures are usually included in these assessments. A correct diagnosis can be ensured by conducting electrophysiological studies and specialized tests that offer additional insights into the illness.
